# *Diphyllobothrium latum* Outbreak from Marinated Raw Perch, Lake Geneva, Switzerland

**DOI:** 10.3201/eid1312.071034

**Published:** 2007-12

**Authors:** Yves Jackson, Roberta Pastore, Philippe Sudre, Louis Loutan, François Chappuis

**Affiliations:** * Geneva University Hospitals, Geneva, Switzerland; † General Directorate of Health, Geneva, Switzerland; ‡ European Programme of Intervention Epidemiology Training, Solna, Sweden

**Keywords:** Diphyllobothrium latum, diphyllobothriasis, outbreak, perch, Switzerland, letter

**To the Editor:** *Diphyllobothrium latum*, a fish tapeworm, has a complex cycle including copepods and freshwater fish as intermediate hosts. Humans are infected by eating raw or undercooked fish meat. Clinical consequences of human infection are generally absent or mild, although anemia due to vitamin B12 deficiency was described in Scandinavia (*1*). Freshwater fish host the parasite in some lakes of Switzerland, Italy, Scandinavia, northeastern Canada, and South America (*1*–*4*). Lake Geneva, in Switzerland, harbors perch, pike, and char, which are considered to be food delicacies and may act as secondary intermediate hosts. Perch are heavily infested (*5**,**6*). To date, *D. latum* has reportedly caused only sporadic cases in western Europe. One outbreak has previously been described in South Korea after 5 persons ate raw redlip mullet. Identification of the *Diphyllobothrium* species in that outbreak was uncertain (*7*).

Since 2001, medical centers in the lake region have reported an increasing number of human cases. We report, to our knowledge, the first outbreak of *D. latum* infections in this region, which occurred after a wedding party in June 2006. The menu included raw, marinated perch fillets caught the same day in Lake Geneva. After *D. latum* infection was diagnosed in 2 guests, all those who attended (n = 32) were contacted within 4 months after the wedding. Information was collected with a standardized questionnaire on personal characteristics; past infection with *D. latum*; consumption of raw perch during the wedding, raw freshwater fish in the last 5 years, or both; and symptoms or visible proglottids in stools. All participants who ate the raw perch dish during the wedding had a stool sample examined for ova and proglottids at the Laboratory of Parasitology of the Geneva University Hospitals. Species identification relied on egg and proglottid morphologic characteristics and epidemiologic factors.

A confirmed case-patient was defined as a case in a guest who ate raw perch at the wedding and had characteristic eggs or proglottids in stool. A probable case-patient was defined as a person who ate raw perch during the wedding and reported a “tagliatelle-like” worm of varying length in stools, without a history of consumption of raw beef, pork, or other raw fish in the previous 5 years and in the absence of laboratory examination of stool sample. All confirmed case-patients received a single 10-mg/kg dose of praziquantel. Stool examination was repeated after treatment.

Twenty-six wedding guests ate raw marinated perch. Seven confirmed cases and 1 probable case of *D. latum* infection occurred (attack rate 30.8%). Infected persons had a median age of 34 years (range 24–60 years) and were more likely to be female. Microscopic examination showed characteristic eggs in 7 patients’ stools and both eggs and proglottids in 3 patients.

None of the patients reported symptoms within 7 days after the dinner. Two patients remained asymptomatic at interview but both were reporting visible worm segments in stools. Six patients (75%) reported symptoms that started 20–91 days after the wedding (median 56 days). Reported symptoms were diarrhea (6 patients), fatigue (5), abdominal pain (4), nausea (3), loss of weight (2), vomiting (1), or dizziness (1). No patient required urgent medical care or missed work. The mean interval between the wedding and the first observation of visible proglottids in stool was 40 days. Seven patients were treated with a single 10-mg/kg dose of praziquantel with no adverse effects reported. One patient treated herself with albendazole (400 mg/day for 3 days) before she was seen at a hospital. All patients became asymptomatic and had negative stool examination results 2–10 weeks after treatment.

None of the patients reported previous or subsequent consumption of raw freshwater fish. Raw fish preparations such as sushi, sashimi, carpaccio, and ceviche are increasingly popular and are now also prepared with local freshwater fish. These new food habits represent a clear risk factor for human infection (*5**,**7*).

The plerocercoid larvae in the fish muscles are easily missed during food preparation. Nor are local fish systematically inspected, as imported fish are. The role of paratenic hosts (e.g., dogs, foxes) in transmission is not fully understood.

Information given to the public and professionals such as food handlers, restaurant owners, and fishermen is a key measure to promote safer food practices. Avoiding serving preparations of raw freshwater fish or selecting fish that are not intermediate hosts of *D. latum* would decrease parasite transmission. Cooking the fish at 55°C for 5 minutes efficiently kills the larvae. Freezing the fish at −20°C for 24 hours is also efficient. International regulations recommend freezing all fish that are expected to be served raw. Notable exceptions are fish from farm culture or from areas where strong evidence proves no source or cases of infection (European community rules 853/2004 annexe III, available from www.paquethygiene.com/reglement_ce_853_2004/reglements_ce_853_2004_du_parlement_europeen_et_du_conseil_annexe_3_section_8.asp#debut). However, enforcing these rules proves very difficult for food safety administrations.
